# Prophylactic antibiotic use during labor and delivery in China: a nationwide, multicenter, hospital-based, cross-sectional study

**DOI:** 10.1186/s12916-022-02577-w

**Published:** 2022-11-02

**Authors:** Qiguo Lian, Tao Zheng, Xiaona Huo, Jun Zhang, Lin Zhang

**Affiliations:** 1 NHC Key Lab of Reproduction Regulation (Shanghai Institute for Biomedical and Pharmaceutical Technologies), Fudan University, Shanghai, 200237 China; 2 Department of Obstetrics, Xinhua Hospital, School of Medicine, Shanghai Jiao Tong University, Shanghai, 200092 China; 3 Department of Obstetrics, The International Peace Maternity and Child Health Hospital, School of Medicine, Shanghai Jiao Tong University, Shanghai, 200030 China; 4 MOE-Shanghai Key Lab of Children’s Environmental Health, Xinhua Hospital, School of Medicine, Shanghai Jiao Tong University, Shanghai, 200092 China; 5 Shanghai Key Laboratory of Embryo Original Disease, Shanghai, 200030 China

**Keywords:** Prophylactic antibiotic use, Guideline adherence, Delivery, China

## Abstract

**Background:**

Prophylactic antibiotic use during delivery is common in routine obstetric practice to prevent infection globally, especially in low- and middle-income countries. In China, however, little is currently known about the national estimates for prophylactic antibiotic use during delivery. Therefore, we aimed to describe the prevalence of prophylactic antibiotic use and guideline adherence using national data in China.

**Methods:**

This cross-sectional study analyzed a national dataset from the China Labor and Delivery Survey in 2015–2016. The primary outcomes were prophylactic antibiotic use and clinician adherence to WHO recommendations for the prevention and treatment of maternal peripartum infections. We estimated the weighted prevalence of the outcomes with Taylor series linearization and investigated the associated factors of the outcomes with logistic regression.

**Results:**

Of the 72,519 deliveries, the prevalence of antibiotic prophylaxis was 52.0%, varying from 92.8% in Shanxi to 17.3% in Hainan. The prevalence of clinician adherence to the WHO guideline was 79.9%, ranging from 93.4% in Shandong to 50.0% in Shanxi. Prophylactic antibiotic use was associated with cesarean delivery (AOR, 55.77; 95%CI, 25.74–120.86), operative vaginal delivery (AOR, 4.00; 95%CI, 1.64–9.78), preterm (AOR, 1.96; 95%CI, 1.60–2.41), premature rupture of membranes (PROM) (AOR, 2.80; 95%CI, 1.87–4.18), and meconium-stained amniotic fluid (AOR, 1.91; 95%CI, 1.30–2.81) in all deliveries and also episiotomy (AOR, 1.48; 95%CI, 1.02–2.16) in vaginal deliveries. Clinician adherence was positively associated with cesarean delivery (AOR, 5.72; 95%CI, 2.74–11.93) while negatively associated with operative vaginal delivery (AOR, 0.26; 95%CI, 0.11–0.61), PROM (AOR, 0.50; 95%CI, 0.35–0.70), and meconium-stained amniotic fluid (AOR, 0.66; 95%CI, 0.48–0.91) in all deliveries. In vaginal deliveries, clinician adherence was negatively associated with episiotomy (AOR, 0.67; 95%CI, 0.46–0.96) and severe perineal trauma (AOR, 0.09; 95%CI, 0.02–0.44). Besides, clinicians in general hospitals prescribed prophylactic antibiotics more likely (AOR, 2.79; 95%CI, 1.50–5.19) and had a lower adherence (AOR, 0.38; 95%CI, 0.20–0.71) than their peers in maternity hospitals.

**Conclusions:**

We observed that about half of all deliveries in China received antibiotics for prophylaxis, and most deliveries were prescribed according to the WHO guideline. Furthermore, the two prevalence rates for prophylactic antibiotic use and clinician adherence varied widely across provinces of China.

**Supplementary Information:**

The online version contains supplementary material available at 10.1186/s12916-022-02577-w.

## Background

Antibiotic prophylaxis is prescribed to prevent infections, not to cure or treat diseases [1]. Prophylactic antibiotic use during delivery is a common obstetric practice to prevent infections globally, especially in low- and middle-income countries (LMICs). Without antibiotic prophylaxis, an estimated 20–25% of infection (20% of endometritis and 25% of wound infection) following cesarean delivery occurs [2], and the incidence of endometritis following operative vaginal delivery could rise to 16% [3]. Existing evidence shows that the use of prophylactic antibiotics for cesarean delivery, perinatal group B *Streptococcus*, and premature rupture of membranes (PROM) could reduce maternal infections [2, 4] and improve neonatal outcomes [2].

These benefits of antibiotic prophylaxis have led to both a high and varied prevalence of prophylactic antibiotic use during labor and delivery worldwide. For example, more than 40% of pregnant women in the USA are given antibiotics immediately before delivery [5], and the prevalence is much higher in LMICs (up to 90% in India [6] and 98% in Vietnam [7]). In addition, a WHO global survey [8] showed that 31.2% of all women undergoing vaginal delivery received prophylactic antibiotics, and the prevalence was highest in the WHO Western Pacific Region (including Cambodia, China, Japan, the Philippines, and Vietnam) for both spontaneous (78.2%) and operative (89.1%) vaginal delivery. However, in China, national estimates for prophylactic antibiotic use during delivery are still minimal.

There are a lot of concerns over the inappropriate use of antibiotics during labor and delivery. Antibiotic overprescribing may contribute to antibiotic resistance and increased morbidity and lead to adverse outcomes for both the mother and newborn [9, 10]. Antibiotic underprescribing also could cause a higher risk of perineal wound-related infection, endometritis, or clinical sepsis [11]. As one of the top ten global public health threats, antibiotic resistance impedes the achievement of the United Nations Sustainable Development Goals. In response to these concerns, many professional organizations have published evidence-based guidelines to specify the recommended conditions for the appropriate prophylactic antibiotic use, and the WHO guideline entitled “WHO recommendations for prevention and treatment of maternal peripartum infections” [12] is one of them in labor and delivery. The WHO guideline summarized 18 prioritized questions related to the prevention of peripartum infections, including the routine use of minor procedures (e.g., perineal/pubic shaving), antimicrobial agents, and antibiotic prophylaxis for preventing infection (e.g., cesarean section) [12]. However, the practices of prophylactic antibiotic prescribing during delivery have not been well characterized, especially in LMICs [13, 14].

To address these research gaps, we analyzed national data from 94 Chinese hospitals to describe the prevalence of prophylactic antibiotic use and clinician adherence to the WHO guideline in 2015 [12], as well as the associated factors.

## Methods

### Study design and population

In this cross-sectional study, we used data from the China Labor and Delivery Survey (CLDS) between March 1, 2015, and December 31, 2016. The CLDS is a national, multicenter, hospital-based, cross-sectional survey to collect the labor and delivery data of new births in China. Using a stratified multistage sampling design to permit a representative sample, the CLDS selected 112 hospitals with at least 1000 annual births from 25 (out of 34) provinces throughout China. The sampling strategy has been described elsewhere [15, 16].

The CLDS data coordination center (DCC) was established to coordinate hospitals, train investigators, and manage the database. For data collection, the CLDS DCC randomly selected 6 weeks within 12 months for hospitals with at least 6000 annual births or 10 weeks within 12 months for hospitals with less than 6000 annual births [15–17]. The trained research nurses retrieved, reviewed, and extracted the data of new births and their mothers from the maternal delivery records under the supervision of the CLDS DCC.

### Inclusion and exclusion criteria

Maternal delivery records for all births within each selected week were eligible and extracted. However, the births at < 24 weeks of gestation or with birthweights of < 500 g were excluded [15–17].

### Ethical approval

The CLDS has been reviewed and approved by the WHO Research Ethics Review Committee and the ethics committees in all participating hospitals. Our data request was approved by the CLDS DCC, and ethics exemption was approved by the Ethics Review Board of the Xinhua Hospital, Shanghai Jiao Tong University School of Medicine (XHEC-C-2015-006), because the data from maternal delivery records in selected hospitals were anonymized and de-identified.

### Measures

This study’s primary outcome was prophylactic antibiotic use during labor and delivery. According to antibiotic prescribing for prophylaxis in each maternal delivery record by the physician in charge, prophylactic antibiotic use was classified as yes (coded as 1), no (coded as 0), or unknown (coded as missing).

The secondary outcome was clinician adherence to the WHO guideline on antibiotic prophylaxis in labor and delivery [12]. We recorded the maternal conditions recommended by the guideline for prophylactic antibiotic prescribing in labor and delivery. According to the WHO guideline during the survey [12], the documented indications of antibiotic prophylaxis include all cesarean deliveries (elective and emergency), preterm PROM, manual removal of the placenta, and severe perineal trauma (third- and fourth-degree perineal lacerations). The WHO guideline [12] also summarized a list of non-indications of antibiotic prophylaxis, including preterm labor with intact membranes (PROM), meconium-stained amniotic fluid, operative vaginal birth, uncomplicated vaginal birth, and episiotomy. We generated a binary variable to denote the clinician adherence status in each maternal delivery according to the WHO guideline [12]: (1) adherence (i.e., a pregnant woman who had at least one indicator received prophylactic antibiotics or a pregnant woman who had no indicator did not receive any prophylactic antibiotics) and (2) non-adherence (i.e., a pregnant woman who had at least one indicator did not receive any prophylactic antibiotics (under-prescription) or a pregnant woman who had no indicator still received prophylactic antibiotics (over-prescription)). Besides, we created a 3-level clinician adherence variable: (1) adherence, (2) over-prescription, and (3) under-prescription.

We also documented other characteristics of the hospitals, including hospital level (i.e., secondary or tertiary), hospital type (i.e., maternity or general), and hospital site (i.e., province).

### Statistical analyses

This study did not have a prospective analysis plan before the survey. To obtain a national estimation of the labor and delivery information, the CLDS DCC calculated a survey weight for each delivery, and the weighting procedure has been described in detail elsewhere [15–17]. We analyzed the data using Stata/SE 15.1 (StataCorp LLC, College Station, TX, USA) with the *svy* prefix commands based on the final person weights and primary sampling units (hospital ID) to account for the complex survey design of the CLDS. The statistical significance level was *P* < 0.05, and all tests were 2-tailed. Complete case analyses were used.

In the descriptive analysis, we calculated weighted prevalence estimates and corresponding 95% confidence intervals (CIs) of prophylactic antibiotic use and clinician adherence to the WHO guideline using Taylor series linearization. We also calculated the weighted prevalence of the two outcome indicators by province and mode of delivery. Besides, we estimated the weighted prevalence of the two outcome indicators for each characteristic studied herein.

In the association analysis, we used logistic regression models to assess the association of potential factors of prophylactic antibiotic use and clinician adherence. Initially, we investigated each factor individually for the two outcome indicators using univariate regression models and calculated odds ratios (ORs) with 95%CIs. Subsequently, we included risk factors studied herein in the final model and estimated the adjusted ORs (AORs) with 95%CIs for each outcome indicator using multivariate regression models.

This study is reported as per the Strengthening the Reporting of Observational Studies in Epidemiology (STROBE) guideline (Additional file 1: Table S1).

### Sensitivity analyses

We also performed additional sensitivity analyses because all cesarean deliveries were recommended for prophylactic antibiotics, and some specific obstetric conditions, including severe perineal trauma and episiotomy, only exist in vaginal deliveries. Briefly, we reproduced all the analyses in vaginal deliveries only and adjusted additional relevant obstetric conditions to evaluate better the factors associated with prophylactic antibiotic use and clinician adherence.

We did not have the data on the indications for antibiotic use, which may limit the interpretation of our results. To distinguish the prophylactic antibiotic prescribing from therapeutic purposes, we generated a subpopulation that had vaginal delivery after excluding the participants with many measured obstetric conditions for antibiotic treatment. The exclusion criteria included sexually transmitted infections in pregnancy, severe gestational hypertension (preeclampsia, eclampsia, and postpartum preeclampsia), uterine rupture or dehiscence, hysterectomy, postpartum hemorrhage, blood transfusion, puerperal infection, amniotic fluid embolism, pulmonary embolism, deep vein thrombosis, and maternal ICU admission. Furthermore, we also estimated the prevalence of antibiotic over-prescription and under-prescription in this subpopulation.

## Results

This study included 96 hospitals with at least a 70% completion rate in 24 provinces of China, and the preliminary sample size was 75,128 [17]. All participants in Heilongjiang province (*n* = 1732) were excluded because the information on antibiotic use was invalid. Of the 73,396 participants from 94 hospitals in 23 provinces, the participants without data on antibiotic use (*n* = 877) were excluded from the analysis, and the missing rate was 1.2% (Additional file 1: Table S2). The final analytical sample size was 72,519 from 94 hospitals in 23 provinces of China.

Of these hospitals, 28 (23.22%) were secondary, and 66 (76.78%) were tertiary; 34 (47.82%) were maternity, and 60 (52.18%) were general. Of these deliveries, 44,220 (60.6%) were delivered by spontaneous vagina delivery, 1285 (1.6%) by operative vaginal delivery, and 26,726 (37.8%) by cesarean delivery.

### Prevalence of prophylactic antibiotic use

**Fig. 1:**
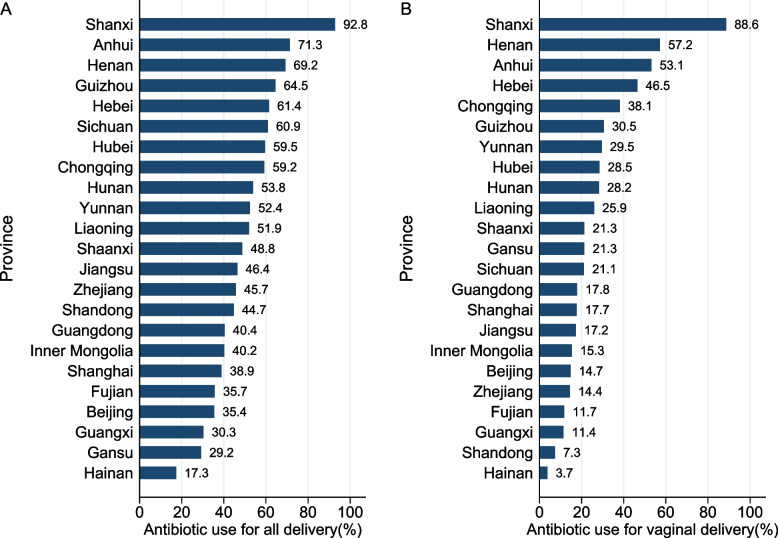
The prevalence of prophylactic antibiotic use across provinces for **A** all delivery and **B** vaginal delivery: China, 2015–2016

In total, 52.0% (95%CI, 45.6–58.3%) of deliveries were given antibiotics for prophylaxis. The prevalence of prophylactic antibiotic use varied substantially across provinces, from 92.8% in Shanxi to 17.3% in Hainan (Fig. 1A). The cesarean delivery rate was 37.8% and varied fivefold across provinces, from 54.6% in Sichuan to 10.2% in Gansu (Additional file 1: Table S3 and Fig. S1). For vaginal deliveries, the prevalence of prophylactic antibiotic use was 27.0%, ranging from 88.6% in Shanxi to 3.7% in Hainan (Fig. 1B); for cesarean deliveries, the prevalence was 93.2%, ranging from 100% in Shanxi to 62.4% in Shanghai (Additional file 1: Table S4).

As shown in Table 1, the univariate analysis revealed that prophylactic antibiotic use was more common among general hospitals (59.3%) than among maternity hospitals (42.1%), was more common among cesarean deliveries (93.2%) and operative vaginal deliveries (57.8%) than among spontaneous vaginal deliveries (26.2%), was more common among preterm births (67.5%) than among term or post-term births (50.5%), was more common among patients with PROM (61.7%) than among those without PROM (50.6%); and was more common among patients with meconium-stained amniotic fluid (62.3%) than among those without meconium-stained amniotic fluid (50.7%). After adjustment, prophylactic antibiotic use was associated with general hospital (AOR, 2.79; 95%CI, 1.50–5.19), cesarean delivery (AOR, 55.77; 95%CI, 25.74–120.86), operative vaginal delivery (AOR, 4.00; 95%CI, 1.64–9.78), preterm (AOR, 1.96; 95%CI, 1.60–2.41), PROM (AOR, 2.80; 95%CI, 1.87–4.18), and meconium-stained amniotic fluid (AOR, 1.91; 95%CI, 1.30–2.81).

**Table 1:** Distribution of characteristics and their associations with prophylactic antibiotic use for all delivery

Characteristics	Unweighted		Weighted			
	Total, no.	Antibiotic use, no.	Proportion, %	Prevalence, %	OR (95%CI, ***P*** value)	AOR (95%CI, ***P*** value)
**Hospital level**						
Secondary	16,836	7624	52.9	53.8	1 [reference]	1 [reference]
Tertiary	55,683	25,486	47.1	50.0	0.86 (0.53–1.39, *P* = 0.535)	0.67 (0.35–1.31, *P* = 0.240)
**Hospital type**						
Maternity	34,678	13,702	42.5	42.1	1 [reference]	1 [reference]
General	37,841	19,408	57.5	59.3	2.00 (1.32–3.01, *P* = 0.001)	2.79 (1.50–5.19, *P* = 0.001)
**Mode of delivery**						
Spontaneous vaginal	44,220	9080	60.6	26.2	1 [reference]	1 [reference]
Operative vaginal	1285	563	1.6	57.8	3.85 (1.22–12.13, *P* = 0.022)	4.00 (1.64–9.78, *P* = 0.003)
Cesarean	26,726	23,340	37.8	93.2	38.58 (19.86–74.91, *P* < 0.001)	55.77 (25.74–120.86, *P* < 0.001)
**Premature rupture of membrane**						
No	62,053	27,006	87.2	50.6	1 [reference]	1 [reference]
Yes	10,398	6069	12.8	61.7	1.57 (1.18–2.10, *P* = 0.003)	2.80 (1.87–4.18, *P* < 0.001)
**Preterm**						
No	64,694	28,321	92.3	50.5	1 [reference]	1 [reference]
Yes	6319	3937	7.7	67.5	2.04 (1.73–2.39, *P* < 0.001)	1.96 (1.60–2.41, *P* < 0.001)
**Meconium-stained amniotic fluid**						
No	65,860	29,523	89.2	50.7	1 [reference]	1 [reference]
Yes	6460	3477	10.8	62.3	1.61 (1.25–2.06, *P* < 0.001)	1.91 (1.30–2.81, *P* < 0.001)

*OR* odds ratio, *AOR* adjusted odds ratio (adjusted for all variables mentioned above), *CI* confidence interval

### Prevalence of clinician adherence to the WHO guideline on antibiotic prophylaxis

**Fig. 2:**
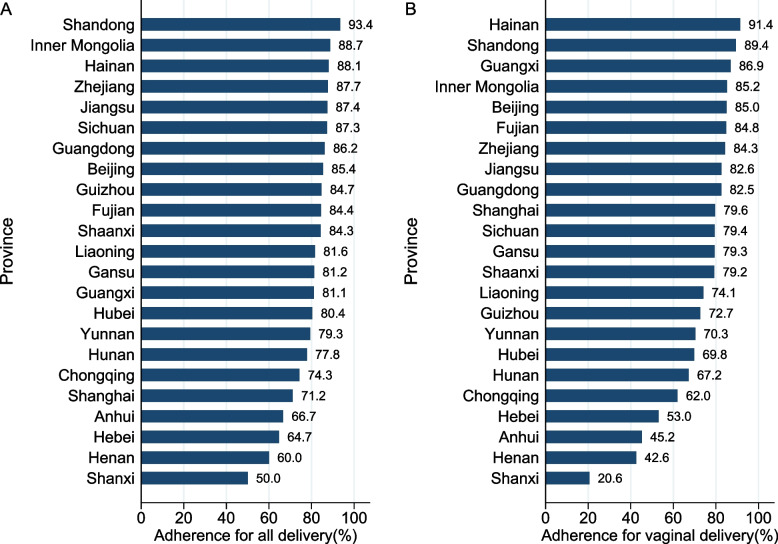
The prevalence of clinician adherence to guidelines across provinces for **A** all delivery and **B** vaginal delivery: China, 2015–2016

In total, 79.9% (95%CI,73.9–84.7%) of all deliveries followed the WHO guideline. Similar to the prevalence of prophylactic antibiotic use, clinician adherence varied substantially across provinces, from 93.4% in Shandong to 50.0% in Shanxi (Fig. 2A). For vaginal deliveries, the prevalence of clinician adherence was 72.1%, ranging from 91.4% in Hainan to 20.6% in Shanxi (Fig. 2B); for cesarean deliveries, the prevalence was 91.4%, ranging from 100% in Shanxi to 62.4% in Shanghai (Additional file 1: Table S5).

As can be seen from Table 2, the univariate analysis indicated that clinician adherence was more common among maternity hospitals (87.2%) than among general hospitals (74.5%); was more common among cesarean deliveries (93.2%) and spontaneous vaginal deliveries (73.0%) than among operative vaginal deliveries (39.1%); was more common among patients without PROM (81.5%) than among those with PROM (68.9%); and more common among patients without meconium-stained amniotic fluid (80.5%) than among those with meconium-stained amniotic fluid (74.6%). Multivariate regression models showed that clinicians were more likely to adhere to the guidelines when the patient was delivered in maternity hospitals (AOR, 2.65; 95%CI, 1.40–5.01) or by cesarean delivery (AOR, 5.72; 95%CI, 2.74–11.93). However, clinician adherence was significantly lower with operative vaginal delivery (AOR, 0.26; 95%CI, 0.11–0.61), PROM (AOR, 0.50; 95%CI, 0.35–0.70), and meconium-stained amniotic fluid (AOR, 0.66; 95%CI, 0.48–0.91).

**Table 2:** Distribution of characteristics and their associations with adherence to the guidelines on antibiotic prophylaxis for all delivery

Characteristics	Unweighted		Weighted			
	Total, no.	Adherence, no.	Proportion, %	Adherence rate, %	OR (95%CI, ***P*** value)	AOR (95%CI, ***P*** value)
**Hospital level**						
Secondary	16,836	14,086	52.9	79.1	1 [reference]	1 [reference]
Tertiary	55,683	44,402	47.1	80.7	1.11 (0.57–2.14, *P* = 0.762)	1.05 (0.56–1.97, *P* = 0.872)
**Hospital type**						
Maternity	34,678	28,802	42.5	87.2	1 [reference]	1 [reference]
General	37,841	29,686	57.5	74.5	0.43 (0.24–0.76, *P* = 0.004)	0.38 (0.20–0.71, *P* = 0.003)
**Mode of delivery**						
Spontaneous vaginal	44,220	34,445	60.6	73.0	1 [reference]	1 [reference]
Operative vaginal	1285	703	1.6	39.1	0.24 (0.09–0.67, *P* = 0.007)	0.26 (0.11–0.61, *P* = 0.002)
Cesarean	26,726	23,340	37.8	93.2	5.07 (2.60–9.89, *P* < 0.001)	5.72 (2.74–11.93, *P* < 0.001)
**Premature rupture of membrane**						
No	62,053	51,577	87.2	81.5	1 [reference]	1 [reference]
Yes	10,398	6874	12.8	68.9	0.50 (0.38–0.66, *P* < 0.001)	0.50 (0.35–0.70, *P* < 0.001)
**Preterm labor**						
No	64,694	53,086	92.3	81.0	1 [reference]	1 [reference]
Yes	6319	4737	7.7	77.9	0.83 (0.58–1.18, *P* = 0.288)	0.77 (0.54–1.10, *P* = 0.145)
**Meconium-stained amniotic fluid**						
No	65,860	53,344	89.2	80.5	1 [reference]	1 [reference]
Yes	6460	5010	10.8	74.6	0.71 (0.54–0.93, *P* = 0.013)	0.66 (0.48–0.91, *P* = 0.011)

*OR* odds ratio, *AOR* adjusted odds ratio (adjusted for all variables mentioned above), *CI* confidence interval

### Sensitivity analyses

After adjusting for additional obstetric conditions, we observed the same consistent associations when examining the prevalence of prophylactic antibiotic use and clinician adherence within vaginal deliveries only (Tables 3 and 4). Patients with episiotomy were more likely to be given prophylactic antibiotics (AOR, 1.48; 95%CI, 1.02–2.16), while patients with severe perineal trauma were not. Clinicians were less likely to adhere to the guidelines when the patient had an episiotomy (AOR, 0.67; 95%CI, 0.46–0.96) or severe perineal trauma (AOR, 0.09; 95%CI, 0.02–0.44).

**Table 3:** Distribution of characteristics and their associations with prophylactic antibiotic use for vaginal delivery only

Characteristics	Unweighted		Weighted			
	Total, no.	Antibiotic use, no.	Proportion, %	Prevalence, %	OR (95%CI, ***P*** value)	AOR (95%CI, ***P*** value)
**Hospital level**						
Secondary	10,634	1974	54.0	29.6	1 [reference]	1 [reference]
Tertiary	34,871	7669	46.0	24.0	0.75 (0.32–1.77, *P* = 0.507)	0.78 (0.35–1.72, *P* = 0.533)
**Hospital type**						
Maternity	22,505	3446	44.5	14.5	1 [reference]	1 [reference]
General	23,000	6197	55.5	37.0	3.47 (1.65–7.28, *P* = 0.001)	3.36 (1.58–7.11, *P* = 0.002)
**Mode of delivery**						
Spontaneous vaginal	44,220	9080	97.5	26.2	1 [reference]	1 [reference]
Operative vaginal	1285	563	2.5	57.8	3.85 (1.22–12.13, *P* = 0.022)	3.36 (1.27–8.86, *P* = 0.015)
**Premature rupture of membrane**						
No	38,110	6323	85.8	23.8	1 [reference]	1 [reference]
Yes	7351	3303	14.2	46.2	2.74 (1.82–4.14, *P* < 0.001)	2.86 (1.79–4.55, *P* < 0.001)
**Preterm labor**						
No	41,672	8212	93.6	25.6	1 [reference]	1 [reference]
Yes	3232	1258	6.4	43.8	2.27 (1.75–2.94, *P* < 0.001)	2.27 (1.80–2.87, *P* < 0.001)
**Meconium-stained amniotic fluid**						
No	41,441	8423	89.6	25.6	1 [reference]	1 [reference]
Yes	3931	1171	10.4	39.3	1.88 (1.33–2.68, *P* = 0.001)	1.82 (1.16–2.85, *P* = 0.010)
**Episiotomy**						
No	33,187	5879	65.3	22.4	1 [reference]	1 [reference]
Yes	12,068	3734	34.7	35.8	1.93 (1.53–2.42, *P* < 0.001)	1.48 (1.02–2.16, *P* = 0.039)
**Perineal laceration degree**						
None, I and II	44,945	9540	99.8	27.0	1 [reference]	1 [reference]
III and IV	72	25	0.2	35.0	1.45 (0.36–5.94, *P* = 0.600)	1.78 (0.58–5.46, *P* = 0.308)

*OR* odds ratio, *AOR* adjusted odds ratio (adjusted for all variables mentioned above), *CI* confidence interval

**Table 4:** Distribution of characteristics and their associations with adherence to the guidelines on antibiotic prophylaxis for vaginal delivery only

Characteristics	Unweighted		Weighted			
	Total, no.	Adherence, no.	Proportion, %	Adherence rate, %	OR (95%CI, ***P*** value)	AOR (95%CI, ***P*** value)
**Hospital level**						
Secondary	10,634	8451	54.0	69.7	1 [reference]	1 [reference]
Tertiary	34,871	26,697	46.0	75.0	1.30 (0.59–2.88, *P* = 0.514)	1.27 (0.60–2.71, *P* = 0.526)
**Hospital type**						
Maternity	22,505	18,570	44.5	83.9	1 [reference]	1 [reference]
General	23,000	16,578	55.5	62.7	0.32 (0.17–0.63, *P* = 0.001)	0.33 (0.16–0.66, *P* = 0.002)
**Mode of delivery**						
Spontaneous vaginal	44,220	34,445	97.5	73.0	1 [reference]	1 [reference]
Operative vaginal	1285	703	2.5	39.1	0.24 (0.09–0.67, *P* = 0.007)	0.32 (0.13–0.77, *P* = 0.012)
**Premature rupture of membrane**						
No	38,110	31,009	85.8	74.7	1 [reference]	1 [reference]
Yes	7351	4120	14.2	56.7	0.44 (0.29–0.68, *P* < 0.001)	0.41 (0.25–0.66, *P* < 0.001)
**Preterm**						
No	41,672	33,061	93.6	73.9	1 [reference]	1 [reference]
Yes	3232	2087	6.4	64.5	0.64 (0.42–0.99, *P* = 0.046)	0.67 (0.42–1.06, *P* = 0.085)
**Meconium-stained amniotic fluid**						
No	41,441	32,365	89.6	73.6	1 [reference]	1 [reference]
Yes	3931	2708	10.4	59.7	0.53 (0.37–0.75, *P* < 0.001)	0.55 (0.36–0.85, *P* = 0.008)
**Episiotomy**						
No	33,187	26,951	65.3	77.0	1 [reference]	1 [reference]
Yes	12,068	8026	34.7	63.0	0.51 (0.41–0.63, *P* < 0.001)	0.67 (0.46–0.96, *P* = 0.028)
**Perineal laceration degree**						
None, I and II	44,945	35,119	99.8	72.6	1 [reference]	1 [reference]
III and IV	72	25	0.2	35.0	0.20 (0.05–0.89, *P* = 0.035)	0.09 (0.02–0.44, *P* = 0.003)

*OR* odds ratio, *AOR* adjusted odds ratio (adjusted for all variables mentioned above), *CI* confidence interval

In the subpopulation without therapeutic indications for an antibiotic prescription (*n* = 41,376), the overall prevalence of prophylactic antibiotic use among vaginal deliveries was 26.1%, ranging from 87.9% in Shanxi to 2.6% in Hainan (Additional file 1: Fig. S2). The overall prevalence of clinician adherence among vaginal deliveries was 73.6%, ranging from 96.7% in Hainan to 23.2% in Shanxi (Additional file 1: Fig. S3). The majority of the non-adherence (92.4%) was antibiotic over-prescription. The overall prevalence of non-adherence by type was 24.4% for over-prescription and 2.0% for under-prescription (Additional file 1: Fig. S3).

## Discussion

### Main findings

This study indicates that the prevalence of prophylactic antibiotic use in China in 2015–2016 was 52.0%, 27.0%, and 93.2% respectively for all deliveries, vaginal deliveries, and cesarean deliveries, with marked provincial variations. In addition, we found that cesarean delivery, operative vaginal delivery, preterm, PROM, meconium-stained amniotic fluid, and episiotomy were associated with an increased risk of prophylactic antibiotic use, and clinicians in general hospitals were more likely to prescribe antibiotics.

This study demonstrates that the prevalence of clinician adherence was 79.9%, 72.1%, and 91.4% respectively for all deliveries, vaginal deliveries, and cesarean deliveries, with large provincial fluctuations. This study also reveals that the vast majority of inappropriate antibiotic prophylaxis was overprescribing. In addition, we observed that cesarean delivery was associated with a higher probability of clinician adherence, while operative vaginal delivery, PROM, meconium-stained amniotic fluid, episiotomy, and severe perineal trauma were associated with a lower likelihood of clinician adherence. Furthermore, clinicians in maternity hospitals were more likely to adhere to the WHO guideline.

### Comparison with previous literature

The prevalence of prophylactic antibiotic use during all deliveries in this study seems much higher than the findings of studies conducted in higher-income countries. Existing research shows that prophylactic antibiotic use during delivery was 30%, 33%, and 39% respectively in the USA [18], Denmark [19], and Canada [20]. Compared with studies from LMICs, the prevalence in this study was similar to a study in Indonesia (47%) [21] and much lower than a study in India (87% for vaginal delivery and 92% for cesarean delivery) [6]. The prevalence of clinician adherence in this study was almost 80%, which was higher than in Indonesia (69%) [21]. The WHO guideline strongly recommends prophylactic antibiotics for cesarean deliveries unless the patient is already receiving an antibiotic regimen with equivalent broad spectrum coverage for existing infectious [1, 12]. The adherence prevalence for cesarean deliveries in this study (93%) was much higher than in Kosovo (66%) and Ecuador (70%) [22].

This study suggested that, among the obstetric conditions, operative vaginal delivery, PROM, meconium-stained amniotic fluid, episiotomy, and severe perineal trauma were associated with lower adherence. It should be noted that the clinician adherence in this study was based on the guidelines in 2015. WHO reviews the recommendations regularly and may update them if new evidence emerges [23]. For example, the WHO recommended not to routinely prescribe antibiotic prophylaxis for operative vaginal delivery in 2015 (recommendation no. 12) [12] but superseded this recommendation in 2021 [23]. Although WHO does not recommend episiotomy for women undergoing spontaneous vaginal birth [24], it was a common practice in this study (32.3%), and the main reason behind it is to reduce potential severe perineal trauma, according to another study carried out in China [25]. Fear of adverse outcomes may contribute to lower adherence to antibiotic prophylaxis guidelines [26]. The obstetric conditions for antibiotic prophylaxis, including PROM, meconium-stained amniotic fluid, and severe perineal trauma, were associated with higher antibiotic prophylaxis use and lower adherence. The main possible reason for the contradictory results is that these obstetric conditions often accompany other symptoms that need to be treated with antibiotics for therapeutic purposes [21].

### Strengths and limitations

This study has several strengths. First, our findings are based on a huge sample from 94 hospitals in 23 provinces; this is the first-ever large-scale national study regarding prophylactic antibiotic use during delivery in China, allowing for national representative and robust statistical results. Second, all delivery outcomes were included, including live birth, stillbirth, fetal death, and neonatal death, which minimized selection bias. Third, the data on antibiotic prophylaxis and obstetric conditions were collected from delivery records and did not rely on self-reported use, which reduced information bias.

This study also has several limitations. First, we only have information on whether an antibiotic was prescribed during delivery, while the data on prescription indicators, drug name, and dosage were unavailable. The antibiotic could be prescribed for prophylactic use, therapeutic use, or both, and we cannot distinguish between them. However, we repeated our analysis in a healthier subpopulation without any measured indicator of the therapeutic use of antibiotics, which minimized the drawback. Second, the retrospective nature of the study design hampered the ability to explore the associated clinician’s characteristics regarding adherence [21]. Besides, we cannot evaluate the temporal trends of prophylactic antibiotic use and clinician adherence in obstetrics practice in China based on the CLDS data. Third, although almost all pregnant women in China have been delivered in hospitals in urban or rural areas since 2014 [27], few deliveries are performed in primary hospitals [16]. Also, all secondary and tertiary hospitals with fewer than 1000 annual deliveries were excluded from our sampling frame. Hence, the study population in this study theoretically may be unable to represent the obstetric population in China. Fourth, the indications for cesarean delivery extracted from delivery records may be influenced by the preferences of clinicians [16].

### Implications

It is critical for clinicians to adhere to the guidelines on antibiotic prescribing in women during and after delivery. Inappropriate antibiotic prescribing (mostly overprescribing) for obstetric conditions has implications on global efforts to contain the emergence of resistant bacteria strains and, consequently, on global health [12]. To reduce the global impact of antibiotic resistance while ensuring access to the best treatment available, WHO published the evidence-based guideline for the prevention and treatment of maternal peripartum infections in 2015 and reviews and updates it at least every 5 years [12, 23].

Improving the clinician’s adherence to the antibiotic prescribing guidelines could reduce inappropriate prophylaxis antibiotic use during delivery because the guidelines were developed to limit the emergence of antibiotic resistance without compromising mother and infant health outcomes [21]. Interventions need to be multifaceted and permanent to guarantee lasting change [26, 28]. First, governments should create appropriate regulations and programs to address antibiotic use and resistance [26]. The China Antimicrobial Surveillance Network monitored national antibiotic resistance and revealed that the 6-year antimicrobial stewardship campaign reduced antibiotic consumption sharply in secondary and tertiary hospitals [29]. Second, the health systems should routinely assess the appropriateness of antibiotic use with the help of independent expert committees [26]. In this study, we found that clinicians in maternity hospitals had higher adherence than their peers in general hospitals, which may reflect a medical culture (e.g., hospital-level antibiotic policy and medical training) difference between the two hospital types. Compared with general hospitals, maternity hospitals may generally adopt rules and regulations on prophylactic antibiotic prescription in stricter enforcement during delivery. Third, academic detailing can promote adherence for targeted clinicians in LMICs [26, 30]. Besides, peer comparison (regularly comparing the inappropriate prescribing rates among clinicians) has a long-term effect partly because it may induce clinicians to make judicious prescribing of antibiotics part of their professional self-image [28].

Besides clinician adherence, reducing cesarean delivery prevalence could also reduce the associated use of antibiotic prophylaxis. In China, about near 39% (37.8% in this study and 38.9% in a previous study [16]) of births were delivered by cesarean delivery, and an absolute 10% reduction to 28.5% (estimated reference) may be considered [16]. Cesarean delivery on maternal request has been a global concern. Apart from previous cesarean delivery (38.2%), maternal request without medical indications (9.8%) was the second-biggest contributor to cesarean delivery [16]. The proportion rose to nearly half of all cesarean deliveries in some areas in southeast China [31], which is much higher than in many other countries [32]. Fear of labor pain, misperceptions of cesarean delivery, and financial incentives for clinicians to perform cesarean delivery remain common in China [33]. Pain relief during vaginal delivery [16], educational package [34], and organizational level audits, training, and financial strategies concerning cesarean delivery [35–37] may help reduce unnecessary cesarean delivery. For every pregnant woman, we should send a more explicit message on the risks and benefits of each mode of delivery and encourage them to work together to reach a shared medical decision in routine obstetric practice [38].

## Conclusions

The overall prevalence was 52.0% for prophylactic antibiotic use and 79.9% for clinician adherence, both with marked provincial variations. Compared with peers in general hospitals, clinicians in maternity hospitals are less likely to prescribe prophylactic antibiotics but more likely to prescribe following the WHO guideline.

## Supplementary Information

**Additional file 1: Table S1.** STROBE checklist for cross-sectional studies. **Table S2.** Missing data rate of prophylactic antibiotics use, stratified by province. **Table S3.** The rate of vaginal and cesarean delivery, stratified by province. **Table S4.** The prevalence of prophylactic antibiotic use, stratified by province and mode of delivery. **Table S5.** The prevalence of adherence to guidelines on antibiotic prophylaxis, stratified by province and mode of delivery. **Figure S1.** The rate of cesarean delivery, stratified by province. **Figure S2.** The prevalence of prophylactic antibiotic use among vaginal deliveries without therapeutic indications. **Figure S3.** The prevalence of adherence to guidelines on antibiotic prophylaxis among vaginal deliveries without therapeutic indications.

## Data Availability

The CLDS data are not publicly available, in accordance with privacy or ethical restrictions. The datasets used and/or analyzed during the current study are available from the corresponding author upon reasonable request.

## Abbreviations

AOR: Adjusted odds ratio
CI: Confidence interval
CLDS: China Labor and Delivery Survey
DCC: Data coordination center
LMICs: Low- and middle-income countries
OR: Odds ratio
PROM: Premature rupture of membranes
STROBE: Strengthening the Reporting of Observational Studies in Epidemiology
