# Supplementary material for: Prophylactic antibiotic use during labor and delivery in China: a nationwide, multicenter, hospital-based, cross-sectional study
Source: BMC Med. 2022 Nov 2;20:391. doi: 10.1186/s12916-022-02577-w (PMC9628083; doi:10.1186/s12916-022-02577-w)
Supplement: Supplementary file 1 — Additional file 1: Table S1. STROBE checklist for cross-sectional studies. Table S2. Missing data rate of prophylactic antibiotics use, stratified by province. Table S3. The rate of vaginal and cesarean delivery, stratified by province. Table S4. The prevalence of prophylactic antibiotic use, stratified by province and mode of delivery. Table S5. The prevalence of adherence to guidelines on antibiotic prophylaxis, stratified by province and mode of delivery. Figure S1. The rate of cesarean delivery, stratified by province. Figure S2. The prevalence of prophylactic antibiotic use among vaginal deliveries without therapeutic indications. Figure S3. The prevalence of adherence to guidelines on antibiotic prophylaxis among vaginal deliveries without therapeutic indications. [file 12916_2022_2577_MOESM1_ESM.docx]

**Table S1. STROBE checklist for cross-sectional studies**

|  | Item No | Recommendation | Relevant content in this paper |
| --- | --- | --- | --- |
| **Title and abstract** | 1 | (*a*) Indicate the study’s design with a commonly used term in the title or the abstract | In Title: ‘A nationwide, multicenter, hospital-based, cross-sectional study’ |
|  |  | (*b*) Provide in the abstract an informative and balanced summary of what was done and what was found | In Methods and Findings section of Abstract. |
| Introduction | | |  |
| Background/rationale | 2 | Explain the scientific background and rationale for the investigation being reported | In para 1-3, Introduction |
| Objectives | 3 | State specific objectives, including any prespecified hypotheses | In para 4, Introduction |
| Methods | | |  |
| Study design | 4 | Present key elements of study design early in the paper | Details of study design are presented in the Study design and Study population section, Methods. |
| Setting | 5 | Describe the setting, locations, and relevant dates, including periods of recruitment, exposure, follow-up, and data collection | In Study design and Study population section, Methods. |
| Participants | 6 | (*a*) *Cohort study*—Give the eligibility criteria, and the sources and methods of selection of participants. Describe methods of follow-up  *Case-control study*—Give the eligibility criteria, and the sources and methods of case ascertainment and control selection. Give the rationale for the choice of cases and controls  *Cross-sectional study*—Give the eligibility criteria, and the sources and methods of selection of participants | Cross-sectional study. In Study design and Study population section, Methods: ‘This study included 96 hospitals with at least 70% response rates in 24 provinces of China, and the preliminary sample size was 75,128. All participants in Heilongjiang province (n=1,732) were excluded because the data on antibiotics use were invalid. Of the 73,396 participants from 94 hospitals in 23 provinces, the participants without data on antibiotics use (n=877) were excluded from the analysis, the missing rate was 1.2%. The final analytical sample size was 72,519.’ |
|  |  | (*b*) *Cohort study*—For matched studies, give matching criteria and number of exposed and unexposed  *Case-control study*—For matched studies, give matching criteria and the number of controls per case | NA  NA |
| Variables | 7 | Clearly define all outcomes, exposures, predictors, potential confounders, and effect modifiers. Give diagnostic criteria, if applicable | Outcomes, exposures, potential confounders are defined and presented in the Measures section. |
| Data sources/ measurement | 8* | For each variable of interest, give sources of data and details of methods of assessment (measurement). Describe comparability of assessment methods if there is more than one group | These are presented in the Measures section. |
| Bias | 9 | Describe any efforts to address potential sources of bias | In Statistical analysis section. |
| Study size | 10 | Explain how the study size was arrived at | In Study design and Study population section. |
| Quantitative variables | 11 | Explain how quantitative variables were handled in the analyses. If applicable, describe which groupings were chosen and why | These are presented in the Measures section. |
| Statistical methods | 12 | (*a*) Describe all statistical methods, including those used to control for confounding | In Statistical analyses section, Methods. |
|  |  | (*b*) Describe any methods used to examine subgroups and interactions | In Statistical analyses section, Methods |
|  |  | (*c*) Explain how missing data were addressed | Analyses were based on complete records of the outcome variables. These are presented in para 2 of Study design and Study population section. |
|  |  | (*d*) *Cohort study*—If applicable, explain how loss to follow-up was addressed  *Case-control study*—If applicable, explain how matching of cases and controls was addressed  *Cross-sectional study*—If applicable, describe analytical methods taking account of sampling strategy | NA |
|  |  | (*e*) Describe any sensitivity analyses | In Sensitivity analyses section. |

| Results | | |  |
| --- | --- | --- | --- |
| Participants | 13* | (a) Report numbers of individuals at each stage of study—eg numbers potentially eligible, examined for eligibility, confirmed eligible, included in the study, completing follow-up, and analysed | In Study design and Study population section. |
|  |  | (b) Give reasons for non-participation at each stage | NA |
|  |  | (c) Consider use of a flow diagram | NA |
| Descriptive data | 14* | (a) Give characteristics of study participants (eg demographic, clinical, social) and information on exposures and potential confounders | In para 1, Results |
|  |  | (b) Indicate number of participants with missing data for each variable of interest | NA |
|  |  | (c) *Cohort study*—Summarise follow-up time (eg, average and total amount) | NA |
| Outcome data | 15* | *Cohort study*—Report numbers of outcome events or summary measures over time | NA |
|  |  | *Case-control study—*Report numbers in each exposure category, or summary measures of exposure | NA |
|  |  | *Cross-sectional study—*Report numbers of outcome events or summary measures | In para 1 in Prevalence of prophylactic antibiotic use and Prevalence of adherence to guidelines on antibiotic prophylaxis section respectively, Results |
| Main results | 16 | (*a*) Give unadjusted estimates and, if applicable, confounder-adjusted estimates and their precision (eg, 95% confidence interval). Make clear which confounders were adjusted for and why they were included | In para 1-2 in Prevalence of prophylactic antibiotic use and Prevalence of adherence to guidelines on antibiotic prophylaxis section respectively, Results |
|  |  | (*b*) Report category boundaries when continuous variables were categorized | NA |
|  |  | (*c*) If relevant, consider translating estimates of relative risk into absolute risk for a meaningful time period | NA |
| Other analyses | 17 | Report other analyses done—eg analyses of subgroups and interactions, and sensitivity analyses | In Sensitivity analyses section. |
| Discussion | | |  |
| Key results | 18 | Summarise key results with reference to study objectives | In para 1-2 in Main findings section, Discussion |
| Limitations | 19 | Discuss limitations of the study, taking into account sources of potential bias or imprecision. Discuss both direction and magnitude of any potential bias | In Strengths and limitations, Discussion |
| Interpretation | 20 | Give a cautious overall interpretation of results considering objectives, limitations, multiplicity of analyses, results from similar studies, and other relevant evidence | Discussion |
| Generalisability | 21 | Discuss the generalisability (external validity) of the study results | In Strengths and limitations, Discussion |
| Other information | | |  |
| Funding | 22 | Give the source of funding and the role of the funders for the present study and, if applicable, for the original study on which the present article is based | In Funding |

*Give information separately for cases and controls in case-control studies and, if applicable, for exposed and unexposed groups in cohort and cross-sectional studies.

**Note:** An Explanation and Elaboration article discusses each checklist item and gives methodological background and published examples of transparent reporting. The STROBE checklist is best used in conjunction with this article (freely available on the Web sites of PLoS Medicine at http://www.plosmedicine.org/, Annals of Internal Medicine at http://www.annals.org/, and Epidemiology at http://www.epidem.com/). Information on the STROBE Initiative is available at www.strobe-statement.org.

**Table S2. Missing data rate of prophylactic antibiotics use, stratified by province**

| **Province** | **Prophylactic antibiotics use, No.** | | | | **Missing data rate, %** |
| --- | --- | --- | --- | --- | --- |
|  | **Yes** | **No** | **Missing** | **Total** |  |
| Total | 33,110 | 39,409 | 877 | 73,396 | 1.2 |
| Anhui | 347 | 311 | 22 | 680 | 3.2 |
| Beijing | 1,676 | 3,172 | 299 | 5,147 | 5.8 |
| Chongqing | 1,897 | 1,171 | 15 | 3,083 | 0.5 |
| Fujian | 1,627 | 3,186 | 56 | 4,869 | 1.2 |
| Gansu | 296 | 839 | 20 | 1,155 | 1.7 |
| Guangdong | 1,347 | 1,765 | 7 | 3,119 | 0.2 |
| Guangxi | 1,551 | 3,574 | 0 | 5,125 | 0 |
| Guizhou | 379 | 209 | 0 | 588 | 0 |
| Hainan | 113 | 541 | 59 | 713 | 8.3 |
| Hebei | 1,997 | 2,823 | 60 | 4,880 | 1.2 |
| Henan | 2,695 | 2,043 | 5 | 4,743 | 0.1 |
| Hubei | 1,125 | 312 | 6 | 1,443 | 0.4 |
| Hunan | 1,867 | 1,659 | 105 | 3,631 | 2.9 |
| Jiangsu | 3,406 | 3,711 | 18 | 7,135 | 0.3 |
| Liaoning | 1,143 | 1,556 | 3 | 2,702 | 0.1 |
| Inner Mongolia | 546 | 813 | 0 | 1,359 | 0 |
| Shandong | 1,430 | 1,566 | 10 | 3,006 | 0.3 |
| Shanxi | 466 | 36 | 0 | 502 | 0 |
| Shaanxi | 1,920 | 2,075 | 15 | 4,010 | 0.4 |
| Shanghai | 557 | 1,699 | 11 | 2,267 | 0.5 |
| Sichuan | 2,050 | 1,389 | 29 | 3,468 | 0.8 |
| Yunnan | 1,514 | 1,256 | 3 | 2,773 | 0.1 |
| Zhejiang | 3,161 | 3,703 | 134 | 6,998 | 1.9 |

**Table S3. The rate of vaginal and cesarean delivery, stratified by province**

| **Province** | **Unweighted No. (weighted proportion, %)** | | |
| --- | --- | --- | --- |
|  | **Vaginal only** | **Cesarean only** | **Vaginal and cesarean** |
| Total | 45,505 (62.2) | 26,726 (37.8) | 72,231 |
| Anhui | 298 (52.9) | 333 (47.1) | 631 |
| Beijing | 3,432 (71.3) | 1,413 (28.7) | 4,845 |
| Chongqing | 1,667 (64.6) | 1,390 (35.4) | 3,057 |
| Fujian | 3,325 (67.1) | 1,470 (32.9) | 4,795 |
| Gansu | 1,062 (89.8) | 72 (10.2) | 1,134 |
| Guangdong | 2,044 (70.9) | 1,067 (29.1) | 3,111 |
| Guangxi | 3,453 (67.4) | 1,670 (32.6) | 5,123 |
| Guizhou | 282 (48.0) | 306 (52.0) | 588 |
| Hainan | 535 (84.1) | 101 (15.9) | 636 |
| Hebei | 3,016 (57.3) | 1,695 (42.7) | 4,711 |
| Henan | 2,887 (62.1) | 1,848 (37.9) | 4,735 |
| Hubei | 570 (50.7) | 858 (49.3) | 1,428 |
| Hunan | 2,195 (62.3) | 1,308 (37.7) | 3,503 |
| Jiangsu | 4,428 (62.8) | 2,677 (37.2) | 7,105 |
| Liaoning | 1,760 (61.5) | 939 (38.5) | 2,699 |
| Inner Mongolia | 942 (69.3) | 417 (30.7) | 1,359 |
| Shandong | 1,717 (59.3) | 1,278 (40.7) | 2,995 |
| Shanxi | 316 (62.9) | 186 (37.1) | 502 |
| Shaanxi | 2,470 (61.7) | 1,501 (38.3) | 3,971 |
| Shanghai | 1,298 (52.7) | 951 (47.3) | 2,249 |
| Sichuan | 1,585 (45.4) | 1,850 (54.6) | 3,435 |
| Yunnan | 1,853 (66.5) | 910 (33.5) | 2,763 |
| Zhejiang | 4,370 (60.4) | 2,486 (39.6) | 6,856 |

Note: Vaginal delivery includes spontaneous vaginal and operative vaginal delivery.

**Table S4. The prevalence of prophylactic antibiotic use, stratified by province and mode of delivery**

| **Province** | **Weighted prevalence of prophylactic antibiotics use, %** | | |
| --- | --- | --- | --- |
|  | **Vaginal only** | **Cesarean only** | **Vaginal and cesarean** |
| Total | 27.0 | 93.2 | 52.0 |
| Anhui | 53.1 | 92.9 | 71.3 |
| Beijing | 14.7 | 86.6 | 35.4 |
| Chongqing | 38.1 | 97.4 | 59.2 |
| Fujian | 11.7 | 84.5 | 35.7 |
| Gansu | 21.3 | 98.4 | 29.2 |
| Guangdong | 17.8 | 96.1 | 40.4 |
| Guangxi | 11.4 | 69.2 | 30.3 |
| Guizhou | 30.5 | 95.8 | 64.5 |
| Hainan | 3.7 | 86.1 | 17.3 |
| Hebei | 46.5 | 81.5 | 61.4 |
| Henan | 57.2 | 88.7 | 69.2 |
| Hubei | 28.5 | 91.4 | 59.5 |
| Hunan | 28.2 | 97.2 | 53.8 |
| Jiangsu | 17.2 | 95.6 | 46.4 |
| Liaoning | 25.9 | 93.7 | 51.9 |
| Inner Mongolia | 15.3 | 96.4 | 40.2 |
| Shandong | 7.3 | 99.1 | 44.7 |
| Shanxi | 88.6 | 100 | 92.8 |
| Shaanxi | 21.3 | 93.6 | 48.8 |
| Shanghai | 17.7 | 62.4 | 38.9 |
| Sichuan | 21.1 | 94.0 | 60.9 |
| Yunnan | 29.5 | 97.7 | 52.4 |
| Zhejiang | 14.4 | 93.2 | 45.7 |

Note: Vaginal delivery includes spontaneous vaginal and operative vaginal delivery.

**Table S5. The prevalence of adherence to guidelines on antibiotic prophylaxis, stratified by province and mode of delivery**

| **Province** | **Weighted adherence prevalence, %** | | |
| --- | --- | --- | --- |
|  | **Vaginal only** | **Cesarean only** | **Vaginal and cesarean** |
| Total | 72.1 | 93.2 | 79.9 |
| Anhui | 45.2 | 92.9 | 66.7 |
| Beijing | 85.0 | 86.6 | 85.4 |
| Chongqing | 62.0 | 97.4 | 74.3 |
| Fujian | 84.8 | 84.5 | 84.4 |
| Gansu | 79.3 | 98.4 | 81.2 |
| Guangdong | 82.5 | 96.1 | 86.2 |
| Guangxi | 86.9 | 69.2 | 81.1 |
| Guizhou | 72.7 | 95.8 | 84.7 |
| Hainan | 91.4 | 86.1 | 88.1 |
| Hebei | 53.0 | 81.5 | 64.7 |
| Henan | 42.6 | 88.7 | 60.0 |
| Hubei | 69.8 | 91.4 | 80.4 |
| Hunan | 67.2 | 97.2 | 77.8 |
| Jiangsu | 85.2 | 96.4 | 88.7 |
| Liaoning | 82.6 | 95.6 | 87.4 |
| Inner Mongolia | 74.1 | 93.7 | 81.6 |
| Shandong | 79.2 | 93.6 | 84.3 |
| Shanxi | 89.4 | 99.1 | 93.4 |
| Shaanxi | 79.6 | 62.4 | 71.2 |
| Shanghai | 20.6 | 1 | 50.0 |
| Sichuan | 79.4 | 94.0 | 87.3 |
| Yunnan | 70.3 | 97.7 | 79.3 |
| Zhejiang | 84.3 | 93.2 | 87.7 |

Note: Vaginal delivery includes spontaneous vaginal and operative vaginal delivery.


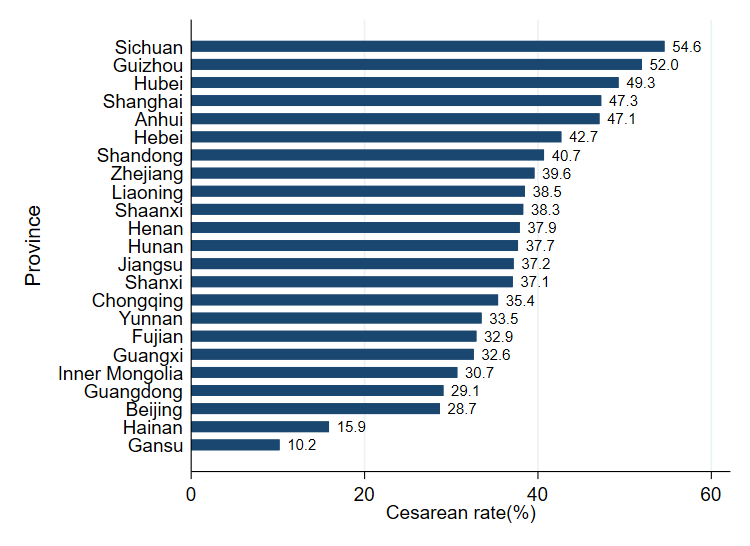


**Figure S1. The rate of cesarean delivery, stratified by province**

**Figure S2. The prevalence of prophylactic antibiotic use among vaginal deliveries without therapeutic indications**

**Figure S3.** **The prevalence of adherence to guidelines on antibiotic prophylaxis among vaginal deliveries without therapeutic indications**
